# MR Imaging Biomarkers to Monitor Early Response to Hypoxia-Activated Prodrug TH-302 in Pancreatic Cancer Xenografts

**DOI:** 10.1371/journal.pone.0155289

**Published:** 2016-05-26

**Authors:** Xiaomeng Zhang, Jonathan W. Wojtkowiak, Gary V. Martinez, Heather H. Cornnell, Charles P. Hart, Amanda F. Baker, Robert Gillies

**Affiliations:** 1 H. Lee Moffitt Cancer Center, Tampa, FL, United States of America; 2 Threshold Pharmaceuticals, South San Francisco, CA, United States of America; 3 Arizona Cancer Center, Tucson, AZ, United States of America; Universidade de São Paulo, BRAZIL

## Abstract

TH-302 is a hypoxia-activated prodrug known to activate selectively under the hypoxic conditions commonly found in solid tumors. It is currently being evaluated in clinical trials, including two trials in Pancreatic Ductal Adenocarcinomas (PDAC). The current study was undertaken to evaluate imaging biomarkers for prediction and response monitoring of TH-302 efficacy in xenograft models of PDAC. Dynamic contrast-enhanced (DCE) and diffusion weighted (DW) magnetic resonance imaging (MRI) were used to monitor acute effects on tumor vasculature and cellularity, respectively. Three human PDAC xenografts with known differential responses to TH-302 were imaged prior to, and at 24 h and 48 hours following a single dose of TH-302 or vehicle to determine if imaging changes presaged changes in tumor volumes. DW-MRI was performed at five b-values to generate apparent diffusion coefficient of water (ADC) maps. For DCE-MRI, a standard clinically available contrast reagent, Gd-DTPA, was used to determine blood flow into the tumor region of interest. TH-302 induced a dramatic decrease in the DCE transfer constant (K^trans^) within 48 hours after treatment in the sensitive tumors, Hs766t and Mia PaCa-2, whereas TH-302 had no effect on the perfusion behavior of resistant SU.86.86 tumors. Tumor cellularity, estimated from ADC, was significantly increased 24 and 48 hours after treatment in Hs766t, but was not observed in the Mia PaCa-2 and SU.86.86 groups. Notably, growth inhibition of Hs766t was observed immediately (day 3) following initiation of treatment, but was not observed in MiaPaCa-2 tumors until 8 days after initiation of treatment. Based on these preclinical findings, DCE-MRI measures of vascular perfusion dynamics and ADC measures of cell density are suggested as potential TH-302 response biomarkers in clinical trials.

## Introduction

The five-year survival rate for pancreatic adenocarcinoma (PDAC) is less than 6% and most survivors are those patients with a surgical option.[1–5] Thus, the majority of pancreatic cancers are treated systemically with chemotherapy, generally gemcitabine (GEM), in combination with other agents. Notably, specific targeted therapies and biologicals such as cetuximab, trastuzamab or bevacizumab have shown little effect against PDAC. A regimen combining 5-Fluorouracil, leucovorin, irinotecan and oxaliplatin (FOLFIRINOX) increased median overall survival (OS) to 11.1 months, compared to 6.8 months for GEM alone.[6] Recently, nab-paclitaxel (ABI-007; nanoparticle-albumin-paclitaxel; Abraxane) has shown significant survival benefit in combination with GEM, and is being considered for front-line status.[7, 8] Even so, response still remains only fleeting and, hence, alternative therapeutic approaches are needed. One promising avenue is to target the cancer’s phenotype, such as hypoxia, which is often observed in pancreatic cancers.[9] This was investigated in Phase I/II (NCT01833546; NCT02047500) trials of TH-302 in combination with GEM, which are complete and a Phase III (NCT01746979) trial of the TH-302 + GEM doublet is underway. Further, a Phase I/II dose escalation trial of TH-302 + GEM + Abraxane triplet combination has recently been opened (NCT02047500).

Tumor hypoxia, characterized by reduced oxygen concentrations, is a significant factor driving tumor physiology and resistance to cancer treatment.[10] The major difference from normal tissue is the irregular structured vasculature matrix found in solid tumors. The microvasculature is characterized by large openings in the endothelium and absence of smooth muscle layer, leading to Enhanced Permeability and Retention (EPR) of macromolecules. These factors result in an unbalanced supply of oxygen and nutrients. This exacerbates the sprouting and inefficient growth of new vessels, which leads to a reduced tumor oxygenation.[11, 12] Although several cancers are known to be hypoxic, pancreatic cancers are known to be profoundly so [13] and intratumoral hypoxia is related to a poor outcome.[14–16] This may be due to the increased levels of the survival factor, HIF-1α [15–18], or selection for defects in the apoptotic machinery.[17] Furthermore, the effect of hypoxia may not be mediated solely by the cancer cells, but may also involve the stroma. Pancreatic cancer is characterized by excessive desmoplastic fibroblasts (stellate cells), whose migration, type I collagen expression, and vascular endothelial growth factor (VEGF) production are all induced by hypoxia.[18] Fundamentally, hypoxia results from an imbalance between oxygen supply and demand. Hypoxic tumor regions are generally resistant to cytotoxic chemotherapy. In part this is due to the direct effects of hypoxia in upregulating cell survival pathways but may also be due to regional perfusion deficits, which result in both hypoxia and sub-therapeutic drug delivery. [14–16, 19, 20] Furthermore, hypoxic tumor cells tend to be quiescent, which also leads to resistance to therapies that are targeted to cell division.[21] Because of the relationship between hypoxia and poor response, one solution for hypoxia-related cancer is introducing hypoxia-activated prodrugs (HAPs), which are selectively activated under extreme hypoxia and deliver high-dose chemotherapy into the hypoxic regions of the tumor with minimal damage in healthy tissue.

The preponderance of hypoxia in PDAC makes it an attractive target for HAPs [22], which have been developed as a family of compounds over the past two decades.[23] HAPs are converted into an active form in reduced oxygen conditions, as they are designed to undergo 1-e^-^ or 2-e^-^ bio-reductions that are reversible in the presence of oxygen. These can be non-specifically catalyzed by a variety of oxoreductases, such as NADPH cytochrome p450 reductase, POR, or inducible nitric oxide synthase, iNOS.[24] The most common class of HAPs is based on 2-nitroimidazoles, exemplified by TH-302. TH-302 is a second-generation HAP that is currently being tested in 12 different clinical trials from Phase I-Phase III. TH-302 releases bromo-isophosphoramide mustard (Br-IPM) under hypoxic conditions, inducing DNA alkylation and cross-linking. Br-IPM can also diffuse into nearby oxygenated regions to prevent tumor progression via “bystander effects”. Both in vitro and in vivo experiments have demonstrated that TH-302 is selectively activated in the tumor hypoxic regions and is also present in the surrounding tissue, but less so in healthy tissues[25]. Pre-clinical studies suggest that TH-302 has significant anti-cancer effect when combined with anti-angiogenic therapies.[26]

Hypoxic regions are often characterized by highly disorganized, dilated and tortuous vessels.[27] This chaotic pattern may result from imbalances of angiogenic mediators such as vascular endothelial growth factor (VEGF) and angiopoietins. Highly permeable tumor vessels may affect cytokines and angiogenic factors, which can dynamically alter the structure of the microvessel walls.[28] Accumulating evidence has already demonstrated that vascular permeability is most likely elevated in hypoxic regions.[27] Thus, evidential and empirical concordance between vessel permeability and hypoxia is established. DCE-MRI uses kinetic modeling of injected contrast reagents to characterize perfusion.[29] It can yield information on the flow, volume, and most importantly the permeability of vessels. Thus it is suitable to assume that DCE-MRI could be a useful surrogate biomarker of hypoxia.[30]

Diffusion-weighted MRI (DW-MRI) detects relatively small changes in tissue structure at the cellular level based on the application of motion sensitive gradients that can trace the movement of water protons. Many preclinical studies have shown that DW-MRI has tremendous potential for monitoring early changes in tumor cellularity that may relate to treatment response.[31] Combination of DCE and DW-MRI may provide more accurate information for early diagnosis and detection of early therapeutic response. [32] Furthermore, these two imaging methods are readily deployed in daily clinic protocols. As such, it is very likely that the results of this study could be easily translated to additional clinic trials.

Previous studies have demonstrated xenograft tumors derived from SU.86.86 human pancreatic cancer cell line are highly vascularized and well-perfused compared to tumors derived from Hs766t cell line.[33] Thus, hypoxic regions observed in Hs766t tumors are larger than those observed in SU.86.86, while tumors derived from Mia PaCa-2 cell line are hypoxic to a medium level. As hypoxia is considered to be a limiting factor for TH-302 activity, it has been experimentally shown that Hs766t-derived tumors are highly sensitive to TH-302 and that SU.86.86-derived tumors are highly resistant, with Mia PaCa-2 exhibit intermediate sensitivity.[34] The present study compared the efficacy of TH-302 in these pancreatic cancer xenograft models with measurements of tumor perfusion and cellularity by using two imaging methods: DW-MRI and DCE-MRI.

## Materials and Methods

### Cell culture

Su.86.86, Hs766t and Mia-PaCa2 cells were acquired from ATCC (Manassas, VA). All cells lines were resuscitated from low passage with all experiments carried out with cells of passage number less than 15. Su.86.86 cells were cultured in RPMI-1640 (Life Technologies) supplemented with 10% FBS (HyClone Laboratories). Hs766t and Mia-PaCa2 cell lines were cultured in DMEM/F12 (Life Technologies) supplemented with 10% FBS and 1% penicillin/streptomycin solution (Sigma, St. Louis, MO). All cell lines were grown at 37°C and 5% CO_2_.

### Xenografts

All procedures were in compliance with the Guide for the Care and Use of laboratory Animal Resources (1996), National Research Council, and approved by the Institutional Animal Care and Use Committee, University of South Florida under the approved protocol R4033. Immunocompromised mice were housed in a clean facility with special conditions that include HEPA filtered ventilated cage systems, autoclaved bedding, autoclaved housing, autoclaved water, irradiated food and special cage changing procedures. Mice were handled under aseptic conditions including the wearing of gloves, gowns and shoe coverings. Mice were sacrificed by inhalation of CO_2_ from a pressurized tank in a mouse chamber.

Female SCID mice of age 5–6 weeks were inoculated with SU.86.86, Hs766t or Mia-PaCa2 cells (5x10^6^) subcutaneously on the left hind leg. Tumors were allowed to grow for an average of three weeks to an average size of ~150 mm^3^, as estimated using electronic calipers and tumor volumes calculated as π/6[(short axis in mm)^2^ x (long axis in mm)]. Mice were then randomized and placed into cohorts and treated with saline (control) or TH-302 (50 mg/kg) injected intraperitoneally. Mice were imaged as described in the magnetic resonance imaging methods section. A total of 34 mice underwent MR imaging studies. The SU.86.86 group consisted of 5 TH-302 treated and 5 control animals; Mia-PaCa2 consisted of 6 TH-302 treated and 5 control animals; Hs766t consisted of 7 TH-302 treated and 6 control animals. No significant animal weight loss was observed during this study. Animals were sacrificed when tumors reached 2000 mm^3^.

### Imaging

Both DCE-MRI and DW-MRI were performed 24 hours prior to the treatment (TH-302 or vehicle), DW-MRI was scheduled 24 hours and 48 hours after treatment. In order to ensure adequate time for contrast agent washout and physiologic recovery, DCE-MRI was only performed 48 hours after treatment. All MRI experiments were performed with a Varian 7 T MRI scanner equipped with a maximum gradient amplitude of 400 mT/m. All animals were anesthetized by inhaled isoflurane (1.5% in O2) at 2.0 LPM and cannulated at the tail vein. A pressure-transducer balloon taped to the animal's chest was used to continuously monitor its respiration rate. The body temperatures were continuously monitored using a rectal fluoroptic thermometer (SAII^®^, SA Instruments, Stony Brook, NY). An external heater was used to maintain body temperature at 37.0 ± 0.2°C and respiratory rate was monitored at a range of 60–90 breaths pre minute during the course of the imaging experiments. The animal was gently secured in a plastic holder and loaded into a 35-mm-ID small-animal imaging Litz coil (Doty Scientific, Columbia, SC, USA). MRI session for both DCE- and DW-MRI scan was approximate 1.5 hours; 0.5 hours for DW-MRI only section.

DCE-MRI protocol includes T_2_-weighted images for anatomical view of tumor, parametric maps of endogenous T_1_ relaxation time and a series of T_1_-weighted images which trace the dynamic change of contrast agent including AIF and wash-in and -out within tumor site. T_2_-weighted images were acquired using a fast spin echo (FSE) pulse sequence with an echo factor of 4, giving an effective TE of 72 ms. Saturation recovery method for T_1_ mapping with five different recovery time (TR) values was acquired prior to injection of the contrast agent (TR = 5000, 2000, 800, 300, 150 ms, TE = 7.2 ms, FOV = 35 x 70 mm, matrix = 128 x 256, spatial resolution = 273 x 273 μm). Nine 1-mm-thick coronal slices were oriented over the tumor and artery to ensure a valid arterial input function (AIF). The animal was tightly secured with tape to avoid motion artifacts. Before, during and after bolus administration of a single dose of CA (0.15 mmol/kg Gd-DTPA), a dynamic series of spin echo T_1_-weighted images (TR = 150 ms, TE = 7.2 ms) were acquired for 35minutes. DCE-MRI data was processed using a general kinetic model as described by Tofts et al. A complete description of these methods has been described previously.[29, 35]

DW-MRI was performed to measure the quantitative ADC values with same geometry as DCE-MRI. Sequence parameters include five b-values: 12, 50, 500, 800, 1500 s/mm^2^ with TR = 1500 ms and TE = 35; Six average were used to reduce the motion artifacts. ADC maps were generated by a home-made Matlab program that fits b-values to the Stejskal-Tanner equation, S = S_0_*EXP(-b*ADC), where S_0_ is the signal amplitude without diffusion weighting and S is the signal amplitude with diffusion weighting.

Regions of interest (ROIs) were determined by T_2_w images with exactly same geometry of DCE- and DW-MRI acquisition. These ROIs were superimposed onto DCE- and DW-MRI data to minimize the variation of anthropic factor. Distribution histograms were obtained for each tumor ROIs of DCE- or DW-MRI. Data are presented as the mean and its standard error. Student’s t tests, ANOVA were used where appropriate. P < 0.05 was considered to be statistically significant.

### Immunohistochemistry

Following completion of therapeutic and MRI imaging protocols, pimonidazole hydrochloride (60 mg/kg; Hypoxyprobe Inc.) was injected I.P. one hour prior to tumor removal, subsequently fixed in 10% formalin, paraffin embedded, and further processed for immunohistochemistry. Tumor cross sections were stained with a rabbit primary antibody against gamma-H2AX (Novus Biologicals, Littleton, CO) and the Ventana OmniMap anti-rabbit secondary antibody (Ventana Medical Systems, Tucson, AZ). Pimonidazole positive tissue was detected using rabbit antisera against pimonidazole hydrochloride (2627; Hypoxyprobe Inc.). The detection system used was the Ventana ChromoMap Kit. Slides were counterstained with hematoxylin & eosin and scanned using the Aperio ScanScope XT.

## Results

**Fig 1:**
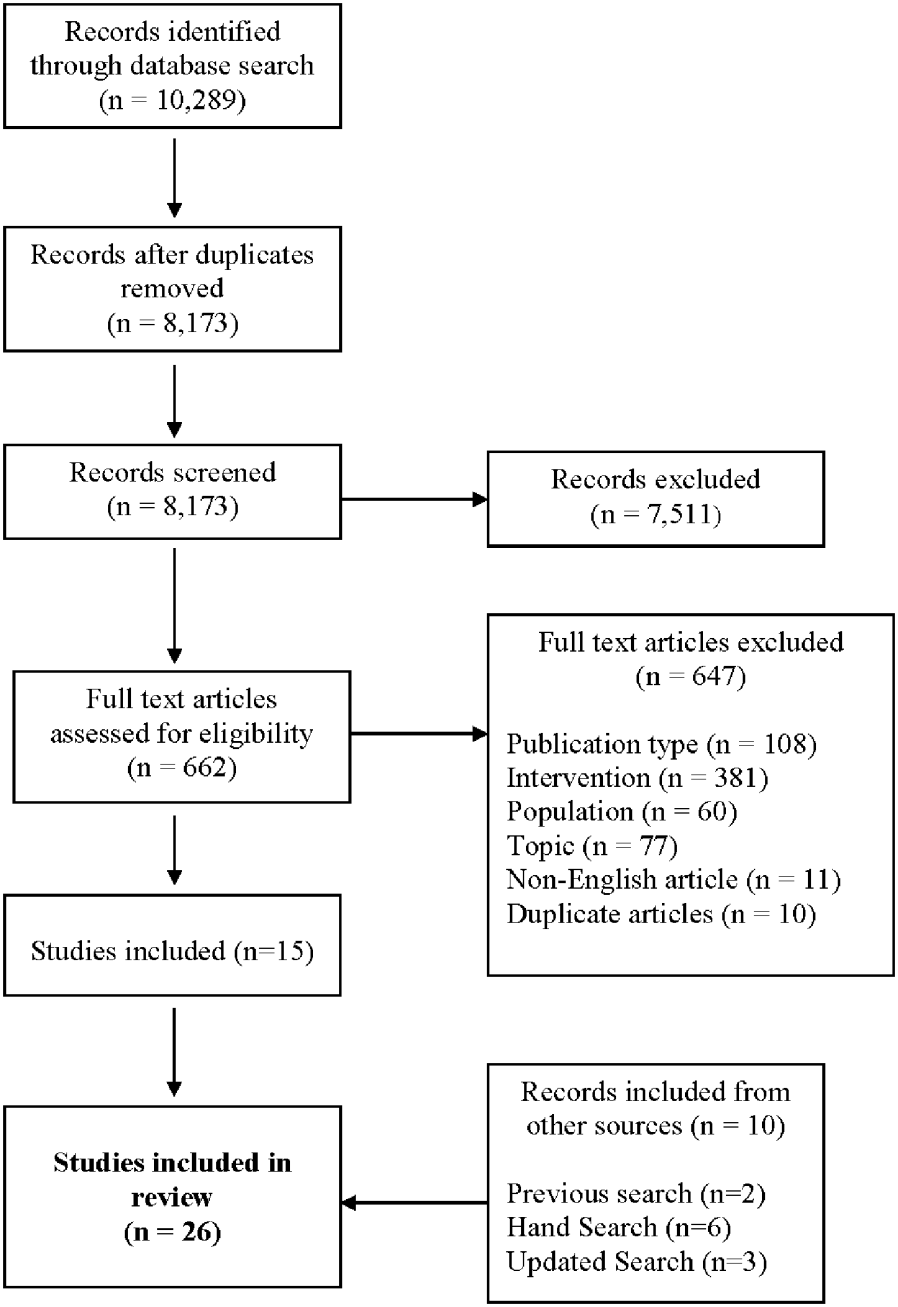
Representative K^trans^ maps for HS766t, Mia PaCa-2 and Su.86.86 tumor type from DCE-MRI (L-R). As demonstrated in the histogram, K^trans^ generated from HS766t and Mia-PaCa-2 tumors were dramatically decreased 48 hours after TH-302 treatment compared to pre-treatment values. There were no significant changes observed for a SU.86.86 mouse at the same time point.

**Fig 2:**
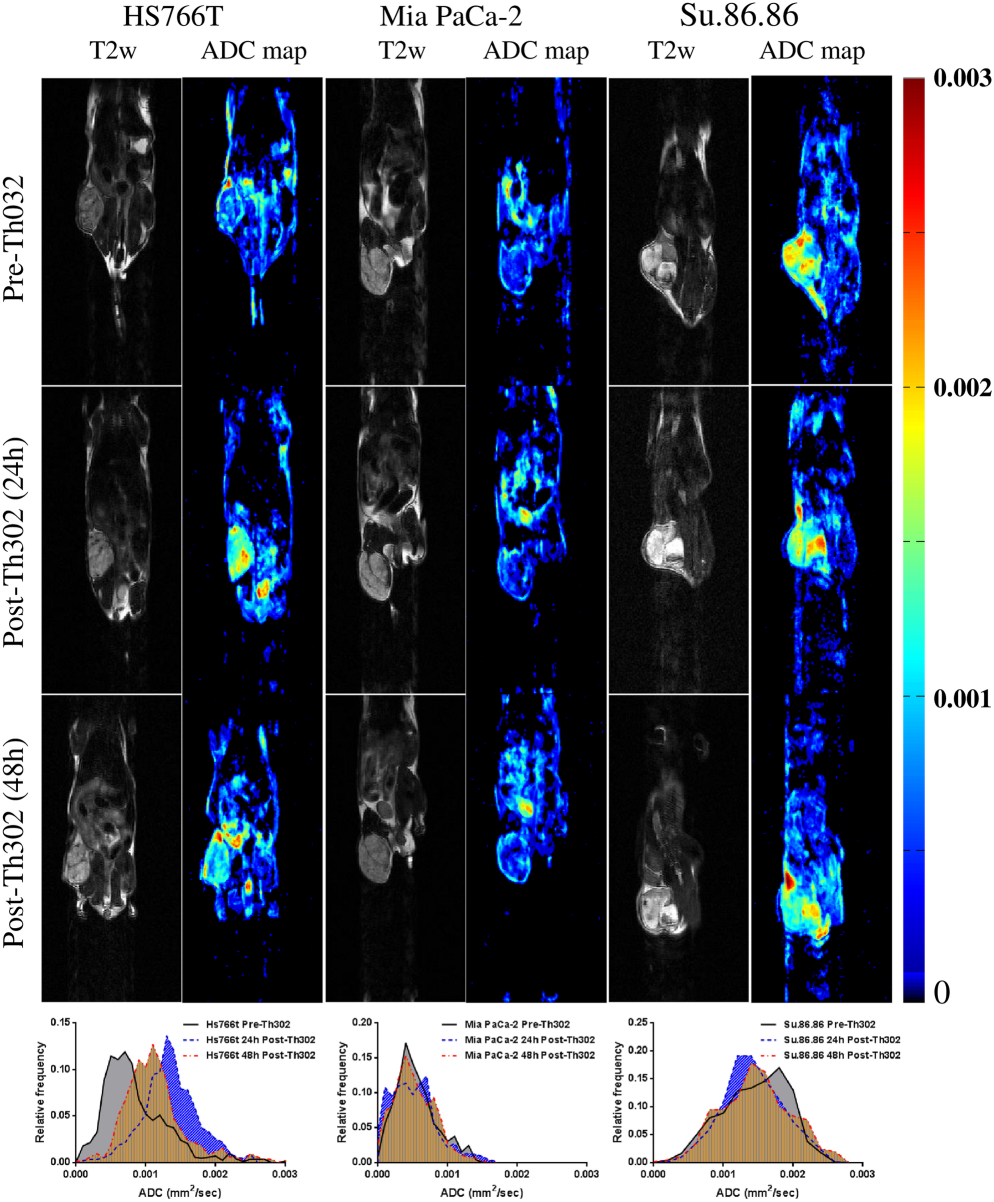
Time-course imaging result of DCE-MRI and DW-MRI. A) Time-courses of normalized mean K^trans^ values. The mean values of normalized K^trans^ decreased 69% for TH-302 treated mice in Hs766t tumors, decreased 46% for Mia PaCa-2 tumors and increased 5% in SU.86.86 tumors. B) Changes in normalized mean tumor ADC values over time. A substantial increase in relative mean ADCs was observed for the TH-302 treated group at post- 24 and 48 hours (29% increasing for 24h, p<0.01; 17% increasing for 48h, p<0.01). MIA PaCa-2 is not statistically significant different between conducted groups (8% increasing for 24h, *P*>0.05; 4% increasing for 48h, p>0.05). For SU.86.86, no significant change was detected by DW-MRI for both TH-302 and control group (3% decreasing for 24h, p>0.05; 0.5% increasing for 48h, p>0.05). The normalization was calculated by the average of the difference of pre- and post-treatment in Th302 group relative to average of the difference of pre- and post-treatment in control group. Error bars stand for standard deviation.

The kinetic parameters of DCE-MRI to quantify tumor perfusion were estimated on a pixel-wise basis by applying the Tofts model as described in [35]. Representative K^trans^ maps for each tumor type are shown in Fig 1. Tumor ROIs (regions of interest) were identified with T_2_-weighted images on all slices, and used to delineate pixels for histogram analyses. As shown by the histograms, the K^trans^ generated from representative Hs766t and Mia-PaCa-2 tumors were dramatically decreased 48 h after TH-302 treatment, as compared to pre-treatment values. There were no significant changes observed for a SU.86.86 tumors at the same time point (Fig 1). Although one individual in the SU.86.86 group had a significant decrease after TH-302 treatment (48 h), the mean changes between comparison groups were not statistically significant. Time-courses of normalized mean K^trans^ values are shown in Fig 2(a). The mean values of normalized K^trans^ decreased 69.2% for TH-302-treated mice in Hs766t tumors, decreased 46.1% for Mia PaCa-2 tumors and increased 4.9% in SU.86.86 tumors. Both changes for Hs766t and Mia PaCa-2 treatment groups were statistically significant (*P*<0.01) when compared to their own control group. In contrast, the mean value increase for SU.86.86 group was not statistically significant (*P* = 0.15). These changes in vascular perfusion correlated with each cell lines therapeutic response to TH-302 [36].

**Fig 3:**
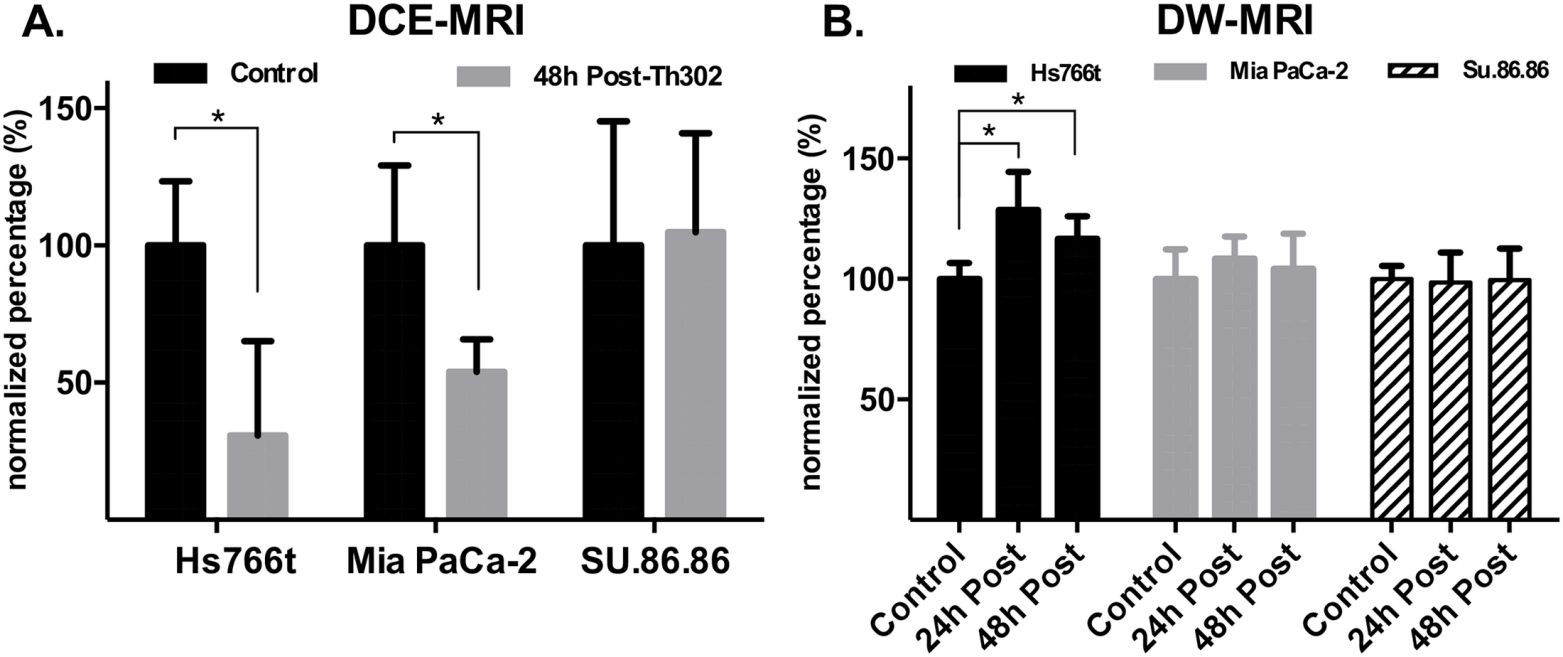
ADC maps and histograms of DW-MRI from representative animals at different time points pre- and post-treatment initiation. A dramatically increased ADCs observed in HS766T, but not in the other two tumor types.

DW-MRI was used to quantify cellularity, and was also used to detect the response of all three tumor xenografts to TH-302. Six averages of DW-acquisitions were used in this study to reduce the potential for motion artifact. ADC maps from representative animals at different time points of pre- and post-treatment are shown in Fig 3. As with the DCE data, T2 images were used to delineate tumor ROI, and circumscribed pixels then used to generate histograms. Changes in normalized mean tumor ADC values over time are presented in Fig 2(b). A substantial increase in relative mean ADCs was observed for the TH-302 treated Hs766t tumors at 24 h and 48 h post-therapy (29% increase for 24h, p<0.01; 17% increase for 48h, p<0.01). For SU.86.86, no significant changes were detected by DW-MRI for both TH-302 and control groups (3% decrease for 24h, p>0.05; 0.5% increase for 48h, p>0.05). Although it appeared that there was an increase in ADC of MiaPaCa-2 at 24 h compared to the vehicle subgroup (8%, P>0.05), these changes were not statistically significantly different from vehicle controls.

**Fig 4:**
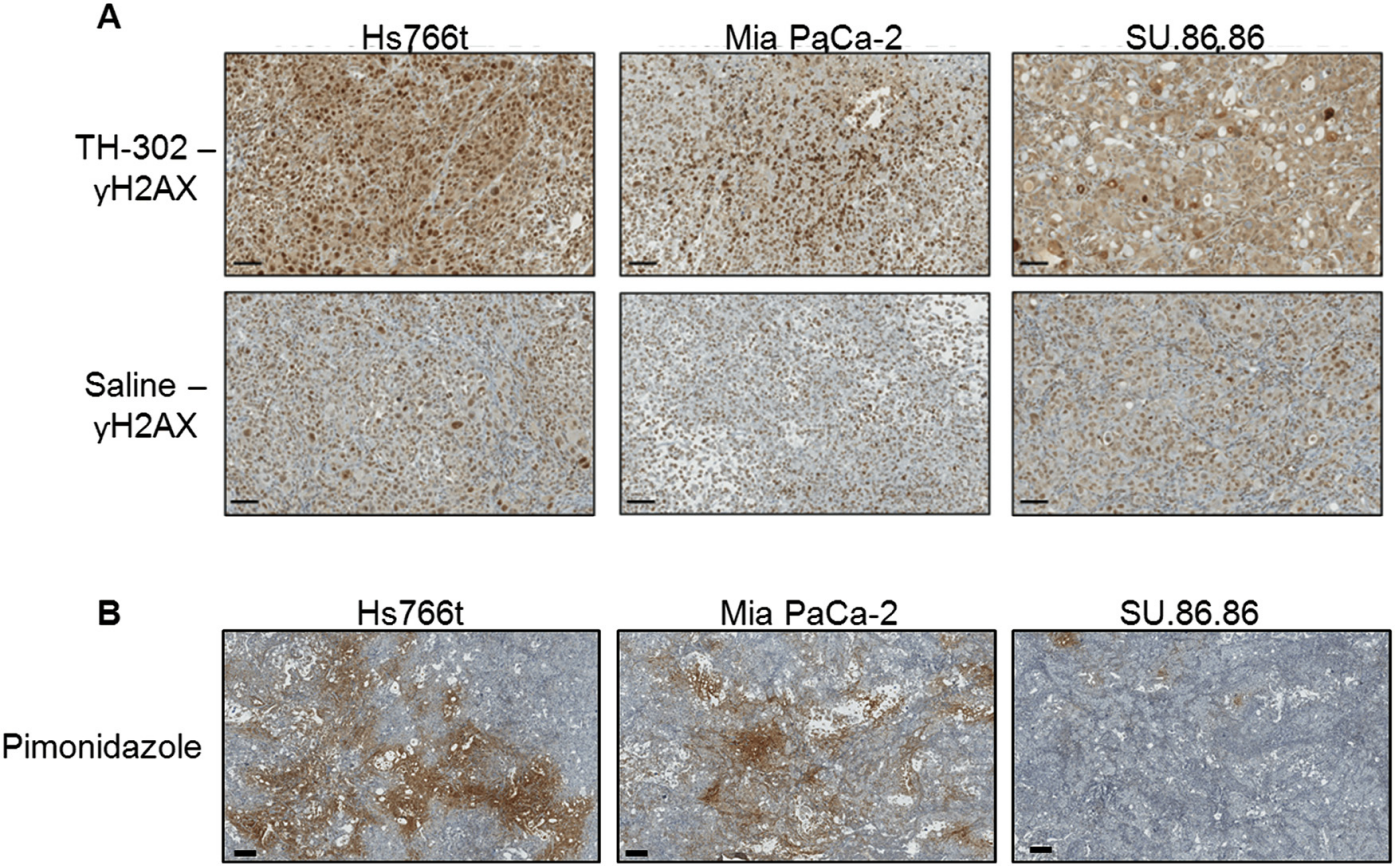
(A) Histological staining of γ-H2AX, a marker for DNA damage response mechanisms, in PDAC tumors pre- and 48hr post-TH-302 treatment (50 mg/kg). Expression of γ -H2AX was increased in TH-302 treated Hs766t and Mia PaCa-2 tumors when compared to saline control. No detectable change in γ-H2AX staining was observed between Su.86.86 treated and saline tumors. (B) Pimonidazole staining as biomarker of physical tumor hypoxia. Pimonidazole Hydrochloride was injected 2hr prior to tumor removal. The extent of hypoxia was greatest in Hs766t tumors and least in SU.86.86 with Mia PaCa-2 tumors moderately hypoxic. These images are representative images of each tumor type. Scale bars = 200 μM.

Statistically significant changes in K^trans^ were observed in Hs766t and Mia PaCa-22 tumors treated with TH-302 for 48 h post-treatment as determined by DCE-MRI. K^trans^ remained unchanged in SU.86.86 tumors (Fig 1). The active effector of TH-302 is a DNA cross-linking agent previously shown to increase nuclear γ-H2AX, a key biomarker for DNA damage response pathways, in vivo following as little as 6 hours post TH-302 therapy in pancreatic tumor models [37], implying early changes in vascular permeability observed in this study may be a consequence of TH-302 cytotoxicity. To draw a relationship between permeability changes and TH-302 activity, tumor cross sections from all three tumor groups were immunohistochemically evaluated for γ H2AX expression (Fig 4A). Nuclear γ -H2AX was increased in TH-302 treated Hs766t and Mia PaCa-2 tumors when compared to the saline control. No detectable change in γ-H2AX staining was observed between Su.86.86-treated and saline-treated tumors. The main determinant of TH-302 activation and efficacy is tumor hypoxia. Immunohistochemical detection of Pimonidazole, a marker of physical hypoxia, showed greater staining in TH-302 sensitive Hs776t and Mia PaCa-2 tumors compared to TH-302 resistant SU.86.86 tumors (Fig 4B) supporting γ-H2AX expression patterns following TH-302 treatment. These data support early changes in Hs766t and Mia PaCa-2 tumor perfusion at 48 hours could possibly be due to the cytotoxic effects of TH-302.

## Discussion

The efficacy of TH-302 is influenced by multiple factors, such as vessel patency and the level of hypoxia in a given region. [38] Having the ability to measure changes in these parameters over time in response to activated TH-302, is critical. Such changes can be used to evaluate therapeutic response. Current non-imaging or invasive methods are limited for in vitro use, while imaging biomarkers can give quantitative measurements of tumor progression or regression, without significantly altering the microenvironment. The two MRI modalities used in this study, DCE-MRI and DW-MRI, can both separately satisfy these requirements based on observable parametric maps, which provide information on vasculature and cellularity, respectively. Furthermore, most tumors are known to be heterogeneous, and especially so in pancreatic cancer. It is therefore of great interest that spatial information can be quantitatively assessed on a pixel-by-pixel basis.[39] Tumor hypoxia is the strongest predictor of tumor response to HAPs and can be measured in patients using PET imaging of 18F-labeled 2-nitroimidazole tracers (F-MISO, FAZA, HX4). However, PET hypoxia imaging is still experimental. In contrast, MR measures of perfusion-diffusion mismatch can be quantitatively related to hypoxia.[24]

This study was focused on identifying and comparing MR imaging biomarkers for detecting early response to TH-302 therapy in sensitive and resistant PDAC tumor models. Prior work from our group had shown that MiaPaCa-2 tumors responded to TH-302 with a slight, but significant, decrease in K^trans^, with no effect on ADC [30]. Those results were confirmed here. Results from DCE-MRI showed a clear decrease of K^trans^ in both Hs766t and Mia PaCa-2 models, which are significantly more hypoxic, compared to SU.86.86 tumors, which did not respond to TH-302 with a change in volume or a change in K^trans^. Consistent with prior work, Hs766t was confirmed by pimonidazole immunohistochemistry (Fig 4) to have the largest hypoxic fraction among these three models; Mia PaCa-2 was intermediately hypoxic and SU.86.86 has minimal hypoxia compared to the other two. [40] So the reduction of permeability could be the consequence of TH-302 activation under the low oxygen conditions resulting in inhibition of VEGF signaling. This is the opposite as seen in non-hypoxic tumor tissues. The degree of permeability changes may directly be related to the degree of hypoxic region. This result corroborates earlier results indicating that TH-302 is most effective in hypoxic tumors [36].

The two MR imaging methods used in this study are fundamentally different: kinetic parameters evaluated from DCE-MRI evaluate vasculature changes, while ADC values fitted by DW-MRI reflect cell density changes. The DCE-MRI reflects the permeability (or flow) changes which may result from cytotoxic effects on the hypoxic tumor cells and reduced VEGF production by activation of TH-302, while DW-MRI is an indirect measurement of the cellular consequences that presumably occur following the vasculature change.[30] Interestingly, we observed that the ADC change was significant only in the most sensitive Hs766t tumors, but not in the other two models. The lack of change in ADC in SU.86.86 is consistent with the lack of effect of TH-302 on tumor growth, and the relative lack of hypoxia in this model. Interestingly, Mia PaCa-2 also did not show a diffusion response to TH-302, despite the fact that tumor growth was inhibited. This was consistent with earlier observations using this tumor line. [30] Notably, the effect of TH-302 therapy on MiaPaCa-2 tumor growth was not apparent until day 8 of treatment, whereas the effect on Hs766t was immediate. Hence, the change in ADC may be able to presage tumor volume changes that occur a day or two later, but not changes 6 days later.

The selection of an arterial input function (AIF) directly affects the kinetic parameters generated by the Tofts model. Factors for AIF estimation may induce errors including partial volume effects, low temporal and spatial resolution, low contrast to noise ratio (CNR) and in many DCE-MRI cases, a poor choice of major arteries in the field of view and motion artifacts. In this study, direct measurement of arterial blood was a challenge, given the respiratory motion during the MR acquisition. Although motion artifacts and low SNR could be reduced by applying a temporal spline-fit function and a spatial Gaussian kernel to smooth the data on pixel-wise analyses, the selection of AIF ROI plays a large role for the final results, as discussed in [35]. This has been the most common issue that affects reproducibility of DCE-MRI, thus large error bar observed in DCE-MRI results. Despite these limitations, DCE-MRI was able to discern differences in TH-302 response, among the three tumor types, in a manner that was consistent with histological findings.

Tumor vasculature is ever changing partly in response to high tumor VEGF concentrations causing aberrant neo-vascularization [41] and solid-phase stress resulting in compression of fragile vasculature [42], both of which contribute to reduced blood flow. In this study, we measured early changes in tumor perfusion following TH-302 treatment. To eliminate the possibility that changes in tumor perfusion following TH-302 are a result of the normal dynamic nature of tumor vasculature, tumor cross sections were histologically stained for nuclear γ -H2AX, a protein involved in DNA Damage Response pathways [43], a measure of TH-302 cytotoxicity. As expected, TH-302 sensitive tumors, Hs766t and Mia PaCa-2, presented with increased nuclear γ -H2AX compared to control tumors. No significant change in tumor perfusion or nuclear γ -H2AX was observed in SU.86.86 tumors, TH-302 resistant. These data support changes in tumor perfusion as measured by DCE-MRI can be used as an early-response biomarker for TH-302 treatment.

In conclusion, our studies suggest that changes in tumor perfusion measured by DCE-MRI may have potential as an early biomarker of TH-302 pharmacodynamics and that DW MRI is a potential biomarker for eventual tumor volume response.
